# Multilevel needs assessment of physical activity, sport, psychological needs, and nutrition in rural children and adults

**DOI:** 10.3389/fpubh.2023.1290567

**Published:** 2023-11-15

**Authors:** Sarah J. Greeven, Andrew M. Medellin, Janette M. Watkins, Cassandra J. Coble, Julia E. Brunnemer, Paola A. Fernández Solá, Sandeep Dutta, James M. Hobson, Justin M. Evanovich, Vanessa M. Martinez Kercher, Kyle A. Kercher

**Affiliations:** ^1^Department of Kinesiology, School of Public Health-Bloomington, Indiana University, Bloomington, IN, United States; ^2^Department of Epidemiology and Biostatistics, School of Public Health-Bloomington, Indiana University, Bloomington, IN, United States; ^3^Program in Neuroscience, College of Arts and Sciences, Indiana University, Bloomington, IN, United States; ^4^Department of Health & Wellness Design, School of Public Health-Bloomington, Indiana University, Bloomington, IN, United States; ^5^Neag School of Education, University of Connecticut, Storrs, CT, United States; ^6^White River Valley School District, Switz City, IN, United States

**Keywords:** human-centered design, multilevel intervention, youth, community-based participatory research, sport-based youth development, psychological needs, sport for development

## Abstract

**Introduction:**

Physical activity yields significant benefits, yet fewer than 1 in 4 youth meet federal guidelines. Children in rural areas from low socioeconomic (SES) backgrounds face unique physical activity contextual challenges. In line with Stage 0 with the NIH Stage Model for Behavioral Intervention Development, the objective of the present study was to conduct a community-engaged needs assessment survey with middle school children and adults to identify perceptions, barriers, and facilitators of physical activity, sport, psychological needs, and nutrition from a multi-level lens.

**Methods:**

A cross-sectional survey data collection was conducted with children (*n* = 39) and adults (*n* = 63) from one middle school community in the Midwestern United States. The child sample was 33% 6th grade; 51% 7th grade and was 49% female. The adult sample was primarily between 30 and 39 years old (70%) and comprised predominantly of females (85%). Multi-level survey design was guided by the psychological needs mini-theory within self-determination theory and aimed to identify individual perceptions, barriers, and facilitators in line with the unique context of the community.

**Results:**

At the individual level, 71.8% of children and 82.2% of the overall sample (children and adults) were interested in new physical activity/sport programming for their school. Likewise, 89.7% of children and 96.8% of adults agree that PA is good for physical health. For basic psychological needs in the overall sample, relatedness was significantly greater than the autonomy and competence subscales. Children’s fruit and vegetable intake were below recommended levels, yet only 43.6% of children were interested in nutritional programming. Conversely, 61.5% indicated interest at increasing leadership skills. At the policy-systems-environmental level, the respondents’ feedback indicated that the condition and availability of equipment are areas in need of improvement to encourage more physical activity. Qualitative responses are presented within for physical activity-related school policy changes.

**Discussion:**

Interventions addressing children’s physical activity lack sustainability, scalability, and impact due to limited stakeholder involvement and often neglect early behavioral intervention stages. The present study identified perspectives, barriers, and facilitators of physical activity, sport, psychological needs, and nutrition in a multi-level context and forms the initial campus-community partnership between scientists and community stakeholders.

## Introduction

Participating in the recommended amount of physical activity (PA) has well-established benefits for physical, psychological, and socioemotional health (1). Despite these benefits, there remains a lack of engagement in PA-related health behaviors among children, particularly those who come from rural and low-socioeconomic status (SES) households (2–4). The disparities between rural and low-SES groups are related to differences in access to PA facilities and resources, as well as existing barriers for transportation, built environment, and socioeconomic status (3, 5, 6). Multi-level (i.e., individual, interpersonal, and community) and multicomponent interventions targeting PA and other positive lifestyle behaviors present an opportunity to reduce inequalities between population groups (7) and are more likely to be successful than single component interventions (8). While many lifestyle interventions have been conducted, few have focused on rural populations and emphasized sustainable multi-level impact on PA-related outcomes, and many have neglected the early stages of human-centered intervention design (7, 9, 10). Instead, many initiatives have imposed researcher’s agendas without adequately taking the time to include stakeholders wants, preferences, and cultural norms. Therefore, while there is general understanding of population-level disparities (e.g., barriers of transportation, cost, lack of feeling welcome), there is a lack of understanding about how the needs manifest in context at the detailed level required to make intervention design decisions.

Numerous lifestyle interventions have been conducted in children, however the outcomes have been mixed and the impact has been limited (8, 11, 12). Community stakeholders are often left feeling frustrated due to short-term emphasis, little long-term benefit, and research teams do not develop the infrastructure to sustain efforts (13, 14). Many interventions fail to scale-up in real world settings (15); as such, early collaboration is recommended between community stakeholders and scientists when designing, implementing, or evaluating interventions (15). Additionally, the existing body of lifestyle intervention literature is limited by a lack of integration of children into the research process. A recent review found less than 1% of published studies of children’s health studies included any form of advice from children regarding their perspectives, preferences, or developmental needs during the research process (16). This lack of inclusion of children in the development of lifestyle interventions fails to take advantage of the recognized unique perspectives and ideas children can contribute that are otherwise unavailable to adult researchers (17, 18). A promising psychological needs-supportive intervention was conducted by Meerits et al. (19) to boost parents’ need-supportive behaviors and support children’s intrinsic motivation for physical activity. Prior literature also supports the use of influential role models as a promising strategy for shaping positive PA experiences for students in PA and physical education (20, 21).

To build on past research and fill in gaps within the existing lifestyle intervention literature, research programs are needed that (1) dedicate significant time to early stages of intervention development and (2) follow systematic/evidence-based intervention development processes. One such approach that addresses these concerns is the National Institute of Health (NIH) Stage Model for Behavioral Intervention Development which aims to support development and testing of interventions at scale in real world settings (22, 23). To progress effectively through the early stages of the NIH Stage Model, a series of preliminary work is essential, including but not limited to using survey methodology that collects input from multiple community stakeholders regarding their wants, preferences, and resources. Collectively, these preliminary steps incorporating elements of community-based participatory research (CBPR) and human-centered design in line with the NIH Stage Model, allow for the foundation of developing a mutually beneficial campus-community partnership aiming to make a lasting impact. We will leverage a campus-community partnership for its myriad benefits, including the valuable opportunity to create collaborative engagements, drawing on diverse perspectives and resources to address real-world challenges and improve the overall quality of research endeavors.

CBPR and the establishment of a campus-community partnership are promising strategies that require essential rapport building to be done in the early stages of the research process. The NIH defines CBPR as programs supporting collaborative interventions that include researchers and community members to address health conditions disproportionately affecting health disparity populations. Relatedly, effective campus-community partnerships require continued investment in shared understanding and usage of common language, rules, expectations, and accountability (24). Partnerships involving campus and community stakeholders, with sport and/or nutrition at the forefront of the collaborations, are growing rapidly and have been associated with the promotion of healthy behaviors (25–27). However, too often is the case where campus-community partnerships are based upon a model that operates with university stakeholders as experts approaching communities as problems to fix (28). Specific examples of inequitable collaboration have been observed in decision-making and short-term programs driven by funding and its accompanying rules (29). Working groups, with stakeholders of diverse identities and roles, operate within a web of active relationships and partnerships with complex dynamics that require effective management and on-going analysis (30–32).

The present study takes a sport-based youth development approach to early-stage intervention development and future testing. Sport-based youth development utilizes sports as a “hook” to promote youth lifestyle development, incorporating physical, social, and psychological components (33). The adoption of sport-based youth developmental programming is driven by its innovative nature, harnessing the inherent benefits and appeal of sports to address and satisfy children’s psychological needs more effectively than conventional exercise initiatives (34). When delivered appropriately, sports foster an environment conducive to self-growth, psychological well-being, and self-esteem in youth (35).

Therefore, we conducted a multilevel needs assessment survey in line with Stage 0 of the NIH Stage Model for Behavioral Intervention Development. Despite knowledge of the PA-based needs of children from the population level, we need to develop specific understanding of how the needs manifest themselves in context at the level of detail needed to make intervention design decisions (36). Further, each rural community is unique and appropriate CBPR requires researcher teams to build rapport and attempt to understand community context prior to intervening. Thus, the objective of the present study was to conduct a needs assessment survey with middle school children and adults to identify perceptions, attributes, barriers, and facilitators of PA/sport, nutrition, and policy-systems-environment (PSE) that are responsive to the community context and preferences. This needs assessment will serve as a starting point for examining the PA-related context with the current middle school partner prior to informing future intervention development and testing.

## Materials and methods

### Conceptual framework

**Figure 1:**
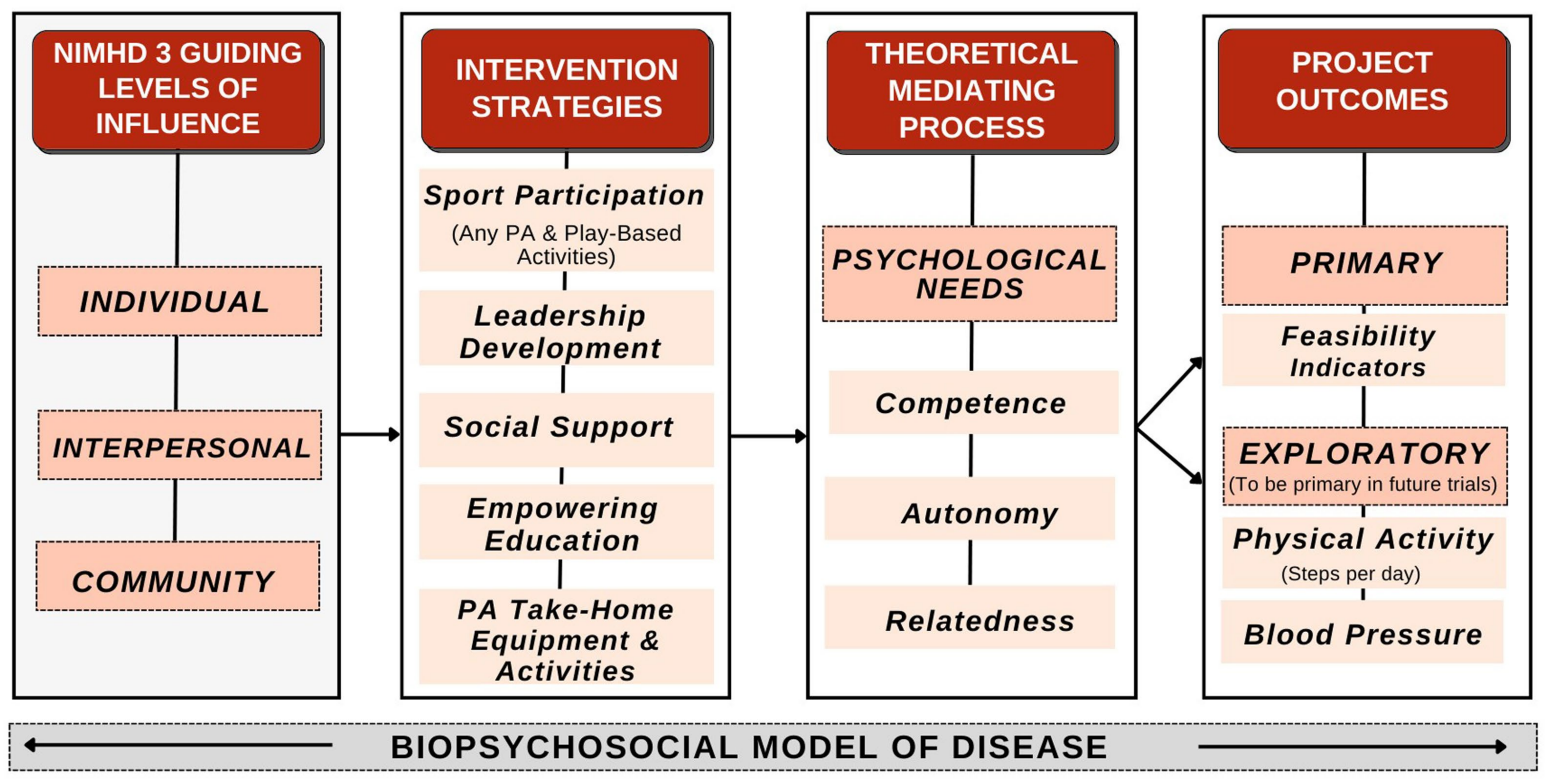
Hoosier sport conceptual framework.

Three complementary theoretical elements were used to guide the present study: (1) the psychological needs satisfaction mini-theory from self-determination theory (37, 38), (2) the National Institute of Minority Health and Health Disparities’ Research Framework (22), and (3) the biopsychosocial model (39). The psychological needs satisfaction mini theory proposes that enhancing a child’s well-being can be achieved by promoting autonomy, competence, and relatedness (40, 41). By incorporating the psychological needs mini theory, we were able to empirically support our examination of the relationship between proposed intervention components, psychological needs, and study outcomes to inform a future exploratory pilot/feasibility study (42–45). The NIMHD Research Framework was used to conceptualize multilevel factors in the survey design and interpretation. The biopsychosocial model guided the interpretation of results as PA, sport participation, nutrition, lifestyle behaviors, and PSE questions/items all exist within a broader biopsychosocial context (39, 46). This integrated conceptual approach is presented in Figure 1 and described further in prior research (36).

### Setting and participants

This prospective cross-sectional cohort study included middle school children (*n* = 39) and adults (defined as parents, adult family members, adult caregivers of children, and/or teachers/administrators) (*n* = 63) from a rural community in Indiana during the 2023 spring semester. The school district serves approximately 800 students from predominantly low-income backgrounds. Of children living in the school district, 96% are non-Hispanic White (47). The district experiences high poverty as 52% of the residents live below the poverty line utilized by the Supplemental Nutrition Assistance Program Education (SNAP-Ed) (48), and the entire school system provides free breakfast and lunch for students with funding from the Department of Education. Moreover, geographical distance between homes, lack of access to physical activity resources, and unhealthy eating habits present significant barriers to positive health behaviors. While there are no specific estimates for physical inactivity among children in the target county, approximately 33% of adults in the Indiana community are physically inactive (47, 49).

Approval was obtained from the district school board and school stakeholders. There were separate inclusion criteria for children and adults. Children had to be enrolled at the middle school (i.e., 6th – 8th grade), attending school, and willing to participate in the survey. This age group was selected based on alignment in the campus-community partnership as well as a significant reduction in PA that has been observed in 6th grade students compared to other ages (50). Adults had to be parents, adult family members, and/or adult caregivers of children currently enrolled at the middle school or employed as teachers/administrators at the middle school and willing to participate in the survey. Adults were included to ensure a multi-level perspective from the target community was gained. There were no exclusion criteria for this study. All participants and their adult caregivers provided informed consent and assent (children). The Indiana University Institutional Review Board approved the study protocol (#18636).

### Data collection procedures

#### Child survey

After receiving consent from parents, we obtained assent from children before they participated in the study to ensure children fully understood the assent document information, including the purpose of the study, study requirements, and potential risks or benefits. Parental consent was collected remotely through an informed consent document distributed through Qualtrics survey software. Child assent and survey administration were conducted through Qualtrics and occurred in-person to increase compliance and understanding. The survey measures included demographics, the Physical Activity Questionnaire for Children (PAQ-C) (51–54), select items from the Expanded Food and Nutrition Education Program (EFNEP) Food and Physical Activity Behaviors Questionnaire (55), and Basic Psychological Needs in Exercise Scale (BPNES) (56, 57). Children were incentivized with a $10 gift card after completing the survey.

#### Parent/guardian/teacher/administrator survey (hereafter referred to as the adult survey)

For the adult survey, we obtained consent and administered the survey remotely. Similar to the child survey, the adult surveys included demographics, questions from the EFNEP Food and Physical Activity Behaviors Questionnaire (55), BPNES (56, 57), as well as additional PSE questions from prior PA research (58). Adults were incentivized with a $10 e-gift card after completing the survey. All surveys included debriefing questions developed by survey methodologists from the Indiana University Center for Survey Research, encouraging participant feedback on survey methodology and assessing potential areas for improvement in future surveys. See the *Measures* section for additional details and see Supplemental materials S1, S2 for the complete child and adult surveys, respectively.

### Measures

#### Physical activity & sport

To assess physical activity and sport participation, participants completed the Physical Activity Questionnaire for Children (PAQ-C) (51, 54). The PAQ-C assessed PA during physical education class, recess, lunch, right after school, evening, weekends, and spare time. The PAQ-C consisted of ten items scored on a 5-point scale ranging from “*no*” activity being a 1 and “*7 times or more*” being a 5. A cumulative score of 1 indicates low PA, whereas a 5 indicates high PA (51, 54). In children, the PAQ-C has demonstrated good internal consistency, acceptable validity, and an adequate Cronbach’s alpha coefficient of 0.72–0.88 (52, 53). One complex PAQ-C question about PA on each of the past 7 days was omitted from the survey to reduce respondent burden.

#### Perceived community barriers, attitudes, and interest in PA/sport programming

Participants responded to two questions about perceived barriers to PA, sport, walking, running, bicycling, and exercising in their community. The response options were: *Not enough areas/places to be physically active*; *Not enough sports teams/groups where I live*; *The places are run-down or do not have enough equipment*; *I cannot get to places to walk, run, bike, or be physically active easily*; *I’m not interested*. These items were adapted from the Barriers to Being Active Quiz (59) to gain an understanding of specific barriers to PA/sport in the community.

Attitudes toward PA were assessed with three questions to get a sense of the respondents’ beliefs in the holistic value of PA for physical health, relationship building, and handling emotions. Interest in new types of PA-related programming was assessed with three questions about PA/sport programming, nutrition programming, and leadership programming. PA/sport and nutrition programming were assessed because those are two of the primary outcome areas of the program. Leadership programming was selected because school stakeholders expressed interest in leadership-specific programming to be included as part of the future intervention design. 4-point Likert scales were used for assessing attitudes and interest in new programming, ranging from “*Not important/interested at all*” to “*Extremely important/interested*.” Respondents moderately or extremely interested in PA/sport programming were categorized as agreeing and interested in new programming.

#### Nutrition

Questions from the EFNEP Food and Physical Activity Behaviors Questionnaire were used to assess dietary intake. Questions covered nutritional behaviors “*over the last 7 days”* and “*yesterday*.” Of the original 30 questions on the questionnaire, the research team selected eight questions for children and ten questions for adults to help ensure the survey will be feasible in terms of respondent burden. Response options allowed participants to select how often they consume various food and drink options. The EFNEP began in 1969, serves all states and U.S. territories, and reaches 450,000 low-income youth and 200,000 low-income adults each year (55, 60). The EFNEP consistently shows more than 90% of adults and 80% of youth report improved nutritional practices (60, 61).

#### Psychological needs satisfaction

Children and adults will rate the satisfaction of their psychological needs in exercise settings with the Basic Psychological Needs in Exercise Scale (BPNES). The BPNES measures psychological needs satisfaction in an exercise context based on autonomy, competence, and relatedness (56, 57, 62). The BPNES consists of 11 items that form a total score and three subscale scores for the degree to that the person experiences satisfaction of each of the three psychological needs. Scores are assessed on a 5-point Likert scale ranging from “*I do not agree at all”* to “*I completely agree*.” Four items assessed autonomy, four for competence, and three for relatedness (62). In adults, the BPNES has demonstrated adequate internal consistency with Cronbach’s alpha coefficients of 0.84 for autonomy, 0.81 for competence, and 0.92 for relatedness, as well as acceptable discriminant and predictive validity (57). The scale scores are also largely unaffected by social desirability bias and have demonstrated stability in repeated measures (57).

#### Policy-systems-environment

The adult survey included questions addressing the PSE level of influence. Questions assessed adults’ interest in PA, nutrition, positive behavioral programming, and perceptions of current school PA policies and interest in new school PA policies. PA environmental questions were informed by past research on perceived environmental variables that may influence PA (58, 63). As PA behaviors exist within an array of settings and levels of influence, questions focused on gaining an understanding of PA behaviors in various settings such as homes, neighborhoods, PA facilities, and parks. For the adult survey, PA-related questions were asked about programming and PA equipment they would like to see offered at the school. There was also a qualitative open-ended question asked to adults: “*Do you think a new WRV school policy should be created to help WRV children be more physically active*?” If respondents answered *yes*, then this item was presented: “*Please describe the general idea or concept that you think a new policy should address.”* These results are presented descriptively and categorized into themes identified by the research team in Table 1. See Supplemental materials S1, S2 for complete versions of the adult and child surveys, respectively.

**Table 1:** Qualitative responses from parents policy-systems-environment questions.

Themes	Illustrative quotes
Reducing sedentary behavior & increasing movement	“More frequent walk/activity breaks. These are still children. They need to be moving more than they are sitting.”
	“Please get our kids out of the seats and moving! They need to get back outside and learn from the world around them.”
	“More fresh air! Or at least let the kids stand up by their desk so they are not sitting so much.”
	“Looking into extend physical education class time, or free time spent outdoors.”
	“Take the lesson outside on nice days. Maybe go for a 10 mins walk outside or something. Will give them a chance to get some vitamin D too and hopefully decrease sickness.”
	“Movement breaks; the opportunity to go out and get fresh air and move your body.”
Tailoring physical activity policy for inclusivity	“The policy should be all inclusive and enticing to all types of children. It should allow for different ranges of abilities to be challenged and rewarded.”
	“Teach the kids how to be more active and engaged in activities.”
	“Recognizing that all children do not have the same learning style and that there is evidence that people learn well by doing hands on activities.”
	“It should address more on being leaders and they should not be scared or intimidated by others because nobody is better than anybody were all human.”
Improving physical education programs & skill development	“Complete overhaul of our programming.”
	“My daughter stated that she only gets 15 min of “recess.” That’s not enough time for kids to get their excess energy out.”
	“Getting kids into physical activities instead of electric activities.”
	“The policy could include ways to get children involved in athletics or activities where you learn to actually compete. Not everyone gets a trophy. We have to instill the work ethic and the desire to be personally better.”
	“I think have PE for an entire semester rather than for one 9 weeks will help with class size and allow each student the same amount of exercise.”
	“The middle school currently does not have the equipment they need like sports balls and their basketball goals are broken.”

### Data analysis

Rather than hypothesis testing, the primary objective of the present study was to identify and describe opportunities, barriers, and facilitators to promote PA and healthy lifestyle behaviors in one rural middle school community. To accomplish this objective, the study team assessed the overall study sample, while from an exploratory standpoint also exploring differences between child and adult respondents for outcomes of interest (e.g., interest in programming). For descriptive statistics, frequencies and percentages were computed for each categorical variable, and means and standard deviations were calculated for continuous variables. Listwise deletion was used for handling missing data as any incomplete observations were dropped from analysis (*n* = 102), which reduced the total sample size to *n* = 39 children and *n* = 63 adults. A sensitivity analysis was conducted by running the analyses on the raw and final samples and the results were highly similar. Analyses were performed in R 4.0.3 (64) and the level of significance was set to alpha = 0.05 in the exploratory analyses.

## Results

### Demographics

A total of 102 respondents completed the survey (overall: *n* = 102; children: *n* = 39; adults: *n* = 63) out of a total 150 students at the middle school (response rate: 39/150 = 26%). The child sample was composed primarily of 6th and 7th grade students (33% 6th grade; 51% 7th grade) and was nearly equally distributed across biological sex (49% female). The adult sample was primarily between 30 and 39 years old (70%) and comprised predominantly of females (85%). The majority of respondents (96%) were Non-Hispanic White, with 37.0% having a high school diploma or lesser qualification, and 74.1% were married or living with a partner. Overall, 94% of participants identified as White. Table 2 provides detailed demographic data for the overall sample.

**Table 2:** Demographics.

Variables	Overall	Children	Adults
*N*	102	39	63
Sex (%)			
Male	26 (27.9%)	18 (46.2%)	8 (14.8%)
Female	65 (69.9%)	19 (48.7%)	46 (85.2%)
Other/prefer not to disclose	2 (2.2%)	2 (5.1%)	–
Age (%) [children / adults]			
10 years old / 30–39	–	3 (7.7%)	38 (70.3%)
11 years old / 40–49	–	11 (28.2%)	13 (24.1%)
12 years old / 50–54	–	11 (28.2%)	2 (3.7%)
13 years old / 55+	–	14 (35.9%)	1 (1.9%)
Race (%)			
White	86 (96.7%)	34 (94.4%)	52 (98.1%)
Asian	–	–	–
Middle Eastern or North	–	–	–
African, Black, or African American	1 (1.1%)	1 (2.7%)	–
American Indian or Alaska Native	1 (1.1%)	1 (2.7%)	–
Native Hawaiian or Other Pacific Islander	–	–	–
Multiracial	1 (1.1%)	–	1 (1.9%)
Ethnicity (%)			
Hispanic	2 (2.2%)	–	2 (3.8%)
Non-Hispanic	87 (97.8%)	36 (100%)	51(96.2%)
Grade (%)			
5th Grade	–	3 (8.3%)	–
6th Grade	–	13 (36.1%)	–
7th Grade	–	20 (55.6%)	–
Education (%)			
High school diploma or less	–	–	20 (37.0%)
Some college, associate’s degree, or higher	–	–	34 (63.0%)
Household income (%)			
$59,999 or less	–	–	23 (41.8%)
$60,000 or more	–	–	23 (41.8%)
Prefer not to answer	–	–	9 (16.4%)
Marital status (%)			
Married or living with a partner	–	–	40 (74.1%)
Single, widowed, or unmarried	–	–	14 (25.9%)
Number of children in household (%)			
1–2 Children	–	–	32 (59.3%)
3+ Children	–	–	22 (40.7%)

Percentages calculated based on number of completed responses for each category.

#### Participant PA/sport and nutrition behaviors

Table 3 displays the types of PA and sports participated in by the child respondents. Our sample, including males and females, had a mean activity score of 2.96 which is consistent with past research that found means of between 2.85 to 3.16 for males and 2.56 to 2.79 for females (51). The score for the PAQ-C is between a 1 and 5 where a score of 1 indicates low PA and a score of 5 indicates high PA. The score of 2.96 shows a moderately active mean score for the child respondents (54). See Supplementary Table S2 for detailed item results for the PAQ-C. In the last 7 days, activities participated in most frequently (7+ times in the past week) by children were jogging and physical conditioning (11 respondents), followed by basketball and baseball/softball (7 respondents).

**Table 3:** Children’s physical activity and sport participation in the past week.

*How often did you do these activities in your free time in the last 7 days?*					
	0 times	1–2 times	3–4 times	5–6 times	7+ times
Bicycling	16	6	4	1	6
Skateboarding	22	6	1	0	2
Walking/hiking	9	10	6	2	6
Tag	11	7	3	5	6
Jogging/running	1	10	11	3	11
Physical conditioning	7	10	5	3	11
Dance	24	4	2	1	2
Rowing	24	4	1	0	2
Swimming	22	5	1	0	6
Baseball/softball	9	10	6	5	7
Football	20	3	4	3	5
Badminton/Tennis/Pickleball	23	6	1	0	2
Soccer	14	14	2	0	4
Volleyball	16	7	3	2	6
Basketball	12	9	6	3	7
Ice Hockey	30	1	0	0	1
Gymnastics	25	1	1	2	2
Martial arts	28	2	2	0	2
Other	5	1	1	1	1

#### Perceived barriers, attitudes, interest in programming

See Table 4 for barriers to PA and Table 5 for attitudes and interest in programming. Overall, participants perceived there were not enough areas to be physically active (47% of respondents) and the places to be active are run-down or do not have enough equipment (40%). For attitudes toward PA, a large majority of respondents believed PA is helpful for physical health (94.1% of respondents), relationship building (91.2%), and handling one’s emotions (87.3%). For interest in new programming at school in the overall sample, respondents were very interested in new PA/sport programming (82.2% of respondents), moderately interested in leadership programming (76.2%), and less interested in nutritional programming (67.0%; 43.6% of children). There were noticeable differences between children and adults in terms of interest in programming as adults were more interested in all three types of programming compared to the child group, but the pattern remained the same with PA/sport being favored, followed by leadership and nutrition programming.

**Table 4:** Perceived barriers toward physical activity in the community.

	Overall (%)	Children (%)	Adults (%)
Sample size	*n* = 102	*n* = 39	*n* = 63
Top 5 problems where you live to be physically active/play sports?			
1. Not enough areas to be physically active.	48 (47.1%)	17 (43.6%)	31 (49.2%)
2. Not enough sports teams where I live.	27 (26.5%)	10 (25.6%)	17 (27.0%)
3. The places to be physically active are run-down or do not have enough equipment.	41 (40.2%)	13 (33.3%)	28 (44.4%)
4. I cannot get to places to be physically active.	17 (16.7%)	7 (17.9%)	10 (15.9%)
5. It costs too much to play sports.	22 (21.6%)	3 (7.7%)	19 (30.2%)
Top 5 problems with where you live to run/bike/walk/exercise?			
1. Not enough places to walk, run, bike, or be physically active where I live.	41 (40.2%)	7 (17.9%)	34 (53.9%)
2. There aren’t enough physical activity groups that I could join.	42 (41.2%)	13 (33.3%)	29 (46.0%)
3. The places to walk, run, bike, or be physically active are run down or have no equipment.	34 (33.3%)	8 (20.5%)	26 (41.2%)
4. I cannot get to places to walk, run, bike, or be physically active easily.	16 (15.7%)	9 (23.1%)	7 (11.1%)
5. I’m not interested in these activities.	11 (10.8%)	4 (10.3%)	7 (11.1%)

**Table 5:** Attitudes and interest in physical activity-related programming.

Attitude toward PA			
	Overall (%)	Children (%)	Adults (%)
Agree that PA is good for physical health	96 (94.1%)	35 (89.7%)	61 (96.8%)
Agree that PA is good for relationship building	93 (91.2%)	32 (82.1%)	61 (96.8%)
Agree that PA is good for handling emotions	89 (87.3%)	31(79.5%)	58 (92.1%)
Interest in new programming			
PA/Sport activities/programming	83 (82.2%)	28 (71.8%)	55 (88.7%)
Nutrition programming	67 (67.0%)	17 (43.6%)	50 (81.9%)
Leadership programming	77 (76.2%)	24 (61.5%)	53(85.5%)

**Figure 2:**
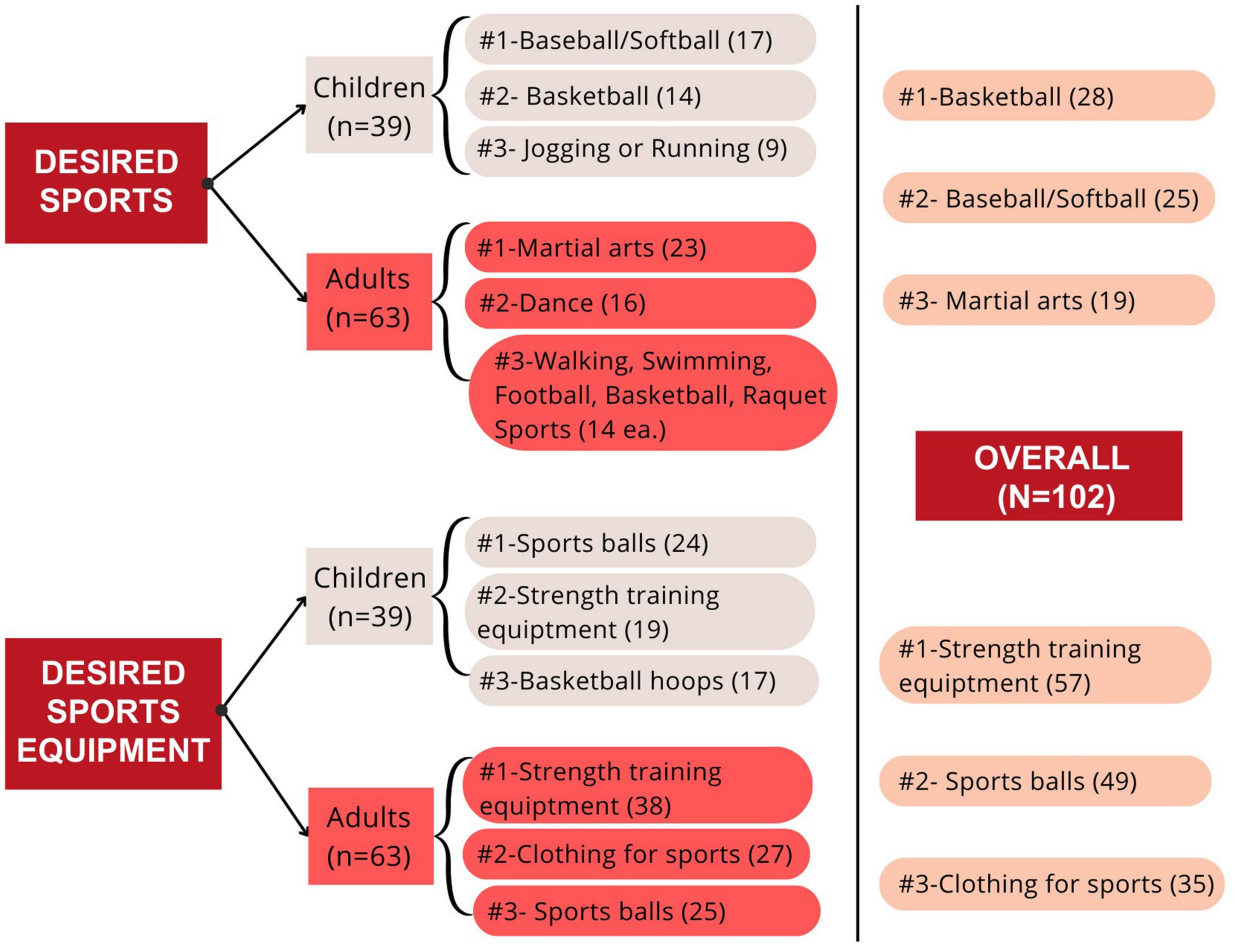
Ranking of top 3 desired sports/activities and equipment to have at school.

The top 3 sports/activities and sports equipment each group would like to see more of at their school are listed in Figure 2. The top sports that respondents would like to see at their school were baseball/softball (children), martial arts (adults), and basketball (overall sample). The top sports equipment desired by respondents were sports balls (children) and strength training equipment (adults and overall sample).

Table 6 illustrates results for nutritional behaviors based on the 7 days recall data from the overall sample, as well as specific breakdowns for child and adult respondents. The data from the child survey highlighted that fruit and vegetable intake were low in the past 7 days. 87.2% of children (34 out of 39 children) reported not consuming a daily serving of fruits and 97.4% did not consume a daily serving of vegetables (38 out of 39 children). 71.8% of children (28 out of 39 children) consumed red/orange vegetables on 0–2 days of the week. 76.9% of children (30 out of 39 children) consumed dark green vegetables on 0–2 days of the week. Children consumed at least one serving of fruit, red/orange vegetable, and dark green vegetables an average of 3.1 days (SD = 1.8), 1.5 days (SD = 1.6), and 1.3 days (SD = 1.5). Additionally, about 89.7% of children reported consuming sweetened beverages in the past week.

**Table 6:** 7 days food recall for child participants.

Nutrition questions	Overall	Children	Adults
How many *days* did you eat fruit?	3.26 (1.71)	3.13 (1.82)	3.34 (1.65)
How many *days* did you eat red or orange vegetables?	2.28 (1.66)	1.54 (1.60)	2.74 (1.54)
How many *days* did you eat dark green vegetables?	2.08 (1.65)	1.33 (1.54)	2.55 (1.54)
How many *days* did you drink sweetened drinks like soda and pop, etc.	1.59 (1.03)	1.39 (0.91)	1.73 (1.09)
How many *times* did you drink sweetened drinks like soda, pop, sports drinks, and energy drinks each day that you drank them?	1.15 (1.18)	1.27 (1.11)	1.08 (1.22)

Results reported as mean (SD).

#### Psychological needs satisfaction

Table 7 displays the cumulative BPNES score as well as the subscale scores for autonomy, competence, and relatedness in the overall, child, and adult samples. For the overall sample, the cumulative score for the BPNES, combining all 11 items, had a mean score of 3.72 (SD = 0.99). The cumulative BPNES scores did not differ significantly between the child and adult groups. Further, the relatedness subscale score was also significantly greater in the adult group compared to the child group (*p* = 0.038). For children, the highest rated item was “*I can easily talk to the people I am physically active with*” [relatedness item]; and the lowest rated item was “*I can do what I need to do to get what I want out of physical activity in my life*” [competence item]. For adults, the highest rated item was “*The relationships with the people I exercise with are friendly*” [relatedness item]; and the lowest rated item was “*I feel exercise is an activity which I do very well*” [competence item].

**Table 7:** Results for basic psychological needs satisfaction in exercise scale^a^.

	Overall	Children	Adults	
Subscale	*n* = 102	*n* = 39	*n* = 63	*p*-value^b^
Autonomy subscale	3.63 (1.17)	3.69 (1.16)	3.60 (1.19)	0.712
Competence subscale	3.43 (1.20)	3.60 (1.00)	3.32 (1.30)	0.231
Relatedness subscale	4.21 (1.11)	3.90 (1.17)	4.39 (1.04)	0.038*
Full scale	3.72 (0.99)	3.73 (0.97)	3.70 (1.00)	0.870

a *t*-test were calculated to examine differences between child and adult participants.

b *p < 0.05. Values presented as mean (SD).

#### PSE

Table 1 provides a summary of qualitative responses from adults to the question “*Do you think a new WRV school policy should be created to help WRV children be more physically active?* If they answered yes, then this item was presented: “*Please describe the general idea or concept that you think a new policy should address.”* 22 adults answered yes to the previous item (16 usable responses) and provided an idea or concept that were then categorized into three major themes: reducing sedentary behavior and increasing movement; tailoring physical activity policy for inclusivity; and improving physical education programs and skill development.

## Discussion

Although many interventions have been conducted to improve PA-related lifestyle behaviors in children, gaps remain in that stakeholders (including children) are often not part of the research process (16) and researchers neglect the early stages of intervention development where critical intervention design decisions are made (36). In line with evidence supporting collaboration between community members and scientists early in the intervention development process (15), this study focused on Stage 0 of the NIH Stage Model to conduct a needs assessment survey with children and adults from a rural Indiana middle school community. The survey focused on identifying perceptions, attributes, barriers, facilitators of PA/sport, nutrition, and PSE that are responsive to the community context. Key findings from this study were (1) a high level of interest in PA/sport programming; (2) basic psychological needs point to promising strengths to build upon and weaknesses to improve; (3) children’s nutritional behaviors were well below recommended levels and they are not very interested in nutrition programming; and (4) adults were engaged in providing ideas for PA-promoting school policy changes. These results are supported by previous work which found school-based PA interventions to be a successful mode for increasing health behaviors (7, 9). Furthermore, this study addresses two major knowledge gaps in literature by (1) establishing a campus-community partnership with a rural school community and (2) providing data to support the success of a multicomponent intervention to increase PA and healthy behaviors (7, 8, 10). The findings from this study will be discussed below and used to inform the next stages of human-centered intervention design prior to pilot/feasibility testing an intervention called Hoosier Sport in the partner middle school. In addition, within the title of our intervention, “Hoosier,” is a prideful term to describe a resident of Indiana.

Similar to nationally representative samples and prior PA literature (65, 66), children in the present study reported lower than recommended levels of physical activity participation. Past research has suggested that the steepest declines in physical activity may occur in 5th and 6th grade children compared to other ages (50). Thus, the present survey results combined with mandatory attendance in middle school point to the middle school as an impactful and promising intervention setting. Child respondents reported participating in many different sports and PAs on a weekly basis, and both children and adults were highly interested in new PA/sport programming being offered at their school (82.2% of overall respondents). Compared to national norms where nearly a quarter of public schools in the United States do not offer sports (23.5%) (67), the middle school in the present study offers 9 sports for girls and 7 sports for boys. Further, data from a nationally representative sample indicate that 33.6% of high poverty schools did not offer sports, compared to only 15.4% of low poverty schools not offering sports (67). Thus, despite being in a high poverty district, the school continues to value and offer a variety of sports to students.

Given the high number of sport/PA offerings from our school partner, it is promising that 72% of children surveyed and 82% of the overall sample are moderately or extremely interested in new sport/PA programming. Additionally, this high level of respondent interest is promising for subsequent intervention design stages, considering that community needs, wants, and preferences will be leveraged rather than preconceived ideas of exercise and sport needs from researchers. Further, an important differentiation between early-stage intervention development in the present study compared to more traditional exercise interventions is that the present study follows a sport-based youth development approach. Based on the high level of interest in new sport/PA programming, our approach looks promising for rooting early-stage intervention development in sport-based youth development and integrating basic psychological needs. Framing Hoosier Sport as a sport-based youth development intervention with strong foundations in basic psychological needs may provide a more promising approach than the more traditional intervention approach of targeting obesity and PA by *requiring* students to do PA for a total of 1 hour per day, which does not capitalize on fun, enjoyability, and positive psychology that may be more accessible through the power of sport. It may be possible that failing to create a fun and enjoyable PA/sport-related context could be a fundamental flaw of many past interventions targeting PA and related lifestyle behaviors.

Our second set of key findings were from the basic psychological needs satisfaction results. In both the child group and adult group, the relatedness subscale was significantly higher than the autonomy and competence subscales. This supports the emphasis by Howie et al. (68) on the potentially positive value of youth sport participation for social health (i.e., relatedness). In terms of future intervention development and evaluation, strong relatedness scores point to co-designing intervention protocol to build on the relatedness psychological need while focusing on improving competency and autonomy. For example, co-design teams may be able to use existing friendships, social supports, or peer-mentoring (i.e., relatedness) to support children in learning new skills (i.e., competence) such as dribbling sports balls in soccer or basketball, learning game rules, or achieving tangible goals in creative school activity programs. Lower competence scores point to an opportunity for competency building activities like SMART goal setting (69) and age-appropriate sport skill developmental activities. As the Hoosier Sport project begins to identify and select intervention strategies, behavioral classification systems targeting each psychological need could be used, as identified in recent research from Ahmadi et al. (70).

While the primary focus of Hoosier Sport is on children, the inclusion of results for adults is also important because children exist within the inseparable context of families and parents, which have a direct influence on child health behaviors (71–73). As these findings are used to develop future intervention protocol, the results from adult respondents are important to support targeting a multi-level intervention strategy with the possibility of influencing adult caregiver (e.g., lifestyle educational materials) and teacher (e.g., professional development training) psychological needs satisfaction. An example of a competency-based strategy that may have a positive impact on both teachers and parents is training on the LET US Play principles for enhancing PA (LET US Play stands for: eliminate **L**ines; avoid **E**limination games; reduce **T**eam sizes; minimize **U**ninvolved kids; adjusting **S**pace, equipment, rules for maximizing PA). If our intervention protocol successfully targets improvements in exercise-related competency in parents and teachers, then children are likely to come in contact with and be influenced by those adults with increased PA-related competency. Further, our preliminary BPNES findings connect the present study to the greater body of psychological needs literature within self-determination theory and provide a concrete example of how the research design has integrated existing theoretical components into various stages of intervention design, implementation, and evaluation. Rigorous design and evaluation are currently lacking within existing sport for social development literature, as many of the programs have been implemented by health and/or non-profit agencies rather than research institutions (68).

The third key finding from the study was that despite nutrition behaviors being below national recommendations (e.g., fruit, vegetable, and sweetened drink intake), children are not very interested in nutrition programming. Only 43.6% of children were interested in new nutrition programming at school compared to 71.8 and 61.5% of children being interested in PA/sport and leadership programming, respectively. To overcome the lack of excitement around nutrition programming, our findings highlight the need for fun and engaging intervention design to target children’s psychological needs satisfaction (i.e., autonomy, competence, relatedness) in a nutritional context. If given the autonomous choice between participating in different types of health programming, children are unlikely to engage in nutrition programming unless it is designed in a fun and engaging way.

Further, the 7 days nutritional recall data from our child respondents highlighted a concerning nutritional situation and potential opportunity for intervention as 87.2% of children reported not consuming a daily serving of fruit; 97.4% of children reported not consuming a daily serving of vegetables; and 89.7% of children reported consuming sweetened beverages at least once in the past week. While the low levels of fruit and vegetable intake are well recognized from a high-level population perspective, these results offer important contextualization and implications in the target community. Rural residents are often under-represented in research, but these results illuminate significant health challenges which help to provide evidence for the rationale of including nutritional education, hands-on nutritional activities, and practical food tasting intervention strategies in our upcoming multicomponent PA-based intervention. The low levels of fruit and vegetable intake in the present study are similar, yet more stark, in comparison to findings from a recent study from the Centers for Disease Control & Prevention (74) which found that in Indiana 43.1% of children did not consume a daily serving of fruit, 53% of children did not consume a daily serving of vegetables, and 66.7% of children consumed sugary beverages at least once weekly. Compared to national norms, Indiana was approximately 5–10% worse with each of the three aforementioned nutritional indicators.

The final key finding based on the open-ended adult PSE responses is the need for new policies to enhance PA and nutrition among WRV children. After expressing interest in the need for improved PA policy, the respondents were then asked to clarify the fundamental concepts that the PA policy should entail. Of the 63 adult respondents, 22 provided responses, revealing three prominent themes: (1) reducing sedentary behavior and increasing movement; (2) customizing PA policies for inclusiveness; and (3) enhancing physical education programs by fostering interpersonal skill development. Regarding inclusivity, the PA policy will cater to diverse abilities and learning styles by promoting active engagement, leadership, and confidence-building activities. In addition, to enhance educational attainment during in-school programming, respondents recommended additional breaks during the day to allow for PA and energy release to maintain focus throughout the school day. Overall, the qualitative responses underscored the awareness and interest of adults in their children’s PA behaviors at school, emphasized an inclusive approach to PA, by recognized the potential value of PA/sport in teaching valuable life skills.

The findings from this study should be interpreted within the study limitations. The present study had a cross-sectional design, and thus causation should not be assumed; rather the study interpretation focused on descriptive statistics and correlation. The study had a non-representative sample, 94% White, but in this case, we were satisfied with that distribution since the community we are collaborating with matches that racial demographic distribution. Although a limitation of this study was that it was convenience sample, we attempted to increase representativeness of the sample by sending the initial study recruitment message to all middle school students through the school administrators, rather than recruiting through athletic groups (e.g., sports teams, at PE class). There was a lack of male representation in the adult sample (85% female); however, the child sample was made up of 49% of respondents identifying as female. Finally, we recognize that inclusivity is a challenge when developing interventions and not all children are interested in group-based physical activity. As such, the results from this survey will help to guide the development of our individualized PA-related goal setting process for future school-based PA interventions. Despite these limitations, the present study highlights a promising CBPR approach to early-stage intervention development that may help lead to more promising interventions putting target communities’ needs, wants, and preferences at the center of the research process. From a practical standpoint, the results from this study will directly inform our next step of intervention design during agenda planning for the human-centered participatory co-design process with children and adults from the partner middle school.

## Data availability statement

The raw data supporting the conclusions of this article will be made available by the authors, without undue reservation.

## Ethics statement

The studies involving humans were approved by Indiana University Institutional Review Board. The studies were conducted in accordance with the local legislation and institutional requirements. Written informed consent for participation in this study was provided by the participants’ legal guardians/next of kin.

## Author contributions

SG: Conceptualization, Data curation, Funding acquisition, Investigation, Methodology, Project administration, Validation, Writing – original draft, Writing – review & editing. AM: Conceptualization, Formal analysis, Methodology, Software, Visualization, Writing – original draft, Writing – review & editing. JW: Writing - reviewing & editing, Visualization, Project administration, Methodology, Supervision. CC: Conceptualization, Funding acquisition, Supervision, Writing – review & editing. JB: Conceptualization, Funding acquisition, Investigation, Methodology, Project administration, Visualization, Writing – review & editing. PF: Conceptualization, Methodology, Visualization, Writing – review & editing. SD: Conceptualization, Investigation, Methodology, Resources, Writing – original draft, Writing – review & editing. JH: Writing - reviewing & editing, Data curation, Visualization, Resources, Project administration, Methodology, Supervision. JE: Investigation, Methodology, Resources, Supervision, Writing – original draft, Writing – review & editing. VM: Conceptualization, Funding acquisition, Investigation, Methodology, Resources, Software, Supervision, Validation, Visualization, Writing – original draft, Writing – review & editing. KK: Conceptualization, Data curation, Formal analysis, Funding acquisition, Investigation, Methodology, Project administration, Resources, Software, Supervision, Validation, Visualization, Writing – original draft, Writing – review & editing.
