# Supplementary material for: Multilevel needs assessment of physical activity, sport, psychological needs, and nutrition in rural children and adults
Source: Front Public Health. 2023 Nov 15;11:1290567. doi: 10.3389/fpubh.2023.1290567 (PMC10684692; doi:10.3389/fpubh.2023.1290567)
Supplement: Supplementary file 1 [file Table_1.DOC]

| **Supplemental Table 1 – Response Rate by Question** | | | |
| --- | --- | --- | --- |
| **Child Survey** | | **Adult Survey** | |
| Question Number | Response Rate | Question Number | Response Rate |
| 1 | 84.44% | 1 | 92.75% |
| 2 | 84.44% | 2 | 92.75% |
| 3 | 84.44% | 3 | 92.75% |
| 3_16_TEXT | 6.67% | 4 | 92.75% |
| 4 | 84.44% | 5 | 92.75% |
| 5 | 77.77% | 6 | 91.30% |
| 6 | 86.67% | Q6_19_TEXT | 5.79% |
| 7 | 86.67% | 7 | 92.75% |
| 8 | 86.67% | Q7_8_TEXT | 10.14% |
| 9 | 86.67% | 8 | 92.75% |
| 10 | 86.67% | Q8_8_TEXT | 2.89% |
| 10_19_TEXT | 11.11% | 9 | 92.75% |
| 11 | 84.44% | Q9_8_TEXT | 4.34% |
| 11_8_TEXT | 8.89% | 10 | 91.30% |
| 12 | 86.67% | 11 | 89.85% |
| 12_8_TEXT | 4.44% | 12 | 91.30% |
| 13 | 86.67% | 13 | 89.85% |
| 13_16_TEXT | 0% | 14 | 33.33% |
| 14 | 86.67% | 15 | 91.30% |
| 15 | 86.67% | 16 | 79.71% |
| 16 | 86.67% | 17 | 75.36% |
| 17 | N/A | 18 | 13.04% |
| 17_19_TXT | 8.89% | 19 | 13.04% |
| 18 | 86.67% | 20 | 91.30% |
| 19 | 86.67% | 21 | 89.85% |
| 20 | 86.67% | 22 | 91.30% |
| 21 | 86.67% | Q23_1 | 91.30% |
| 22 | 86.67% | Q23_2 | 91.30% |
| 23 | 86.67% | Q23_3 | 91.30% |
| 24 | 86.67% | Q23_4 | 91.30% |
| 25 | 86.67% | Q23_5 | 91.30% |
| 26 | 0 | Q23_6 | 91.30% |
| 27 | 83.23% | Q23_7 | 91.30% |
| 28 | 86.67% | Q23_8 | 91.30% |
| 29 | 86.67% | Q23_9 | 89.85% |
| 30 | 86.67% | Q23_10 | 89.85% |
| 31 | 86.67% | Q23_11 | 91.30% |
| 32 | 86.67% | Q24 | 91.30% |
| 33 | 86.67% | Q25 | 91.30% |
| 34 | 86.67% | Q26 | 89.85% |
| 35 | 75.56% | Q27 | 91.30% |
| 36 | 86.67% | Q28 | 91.30% |
| 37 | 80.00% | Q29 | 91.30% |
| 38 | 86.67% | Q30 | 91.30% |
| 39 | 80.00% | Q31 | 78.26% |
| 40 | 80.00% | Q32 | 79.71% |
| 41 | 8.89% | Q33 | 79.71% |
| 42 | 17.78% | Q34 | 79.71% |
| 43 | 22.22% | Q35 | 79.71% |
| 44 | 17.78% | Q36 | 79.71% |
| 45 | 84.44% | Q37 | 79.71% |
| 46 | 17.78% | Q38 | 79.71% |
|  |  | Q39 | 79.71% |
|  |  | Q40 | 79.71% |
|  |  | Q41 | 79.71% |
|  |  | Q42 | 79.71% |
|  |  | Q43 | 78.26% |
|  |  | Q44 | 7.24% |
|  |  | Q45 | N/A |
|  |  | Q46 | 5.79% |
|  |  | Q47 | 8.69% |
|  |  | Q48 | 8.69% |
|  |  | Q49 | 89.85% |
|  |  | Q50 | 4.34% |
