# Supplementary material for: Multilevel needs assessment of physical activity, sport, psychological needs, and nutrition in rural children and adults
Source: Front Public Health. 2023 Nov 15;11:1290567. doi: 10.3389/fpubh.2023.1290567 (PMC10684692; doi:10.3389/fpubh.2023.1290567)
Supplement: Supplementary file 2 [file Table_2.DOC]

**Supplemental Table 2. Item Results for Physical Activity Questionnaire – Child (PAQ-C).**

| Child Survey Question # | PAQ-C  Question # | Composite Score  Child Mean | Cumulative Mean Score |
| --- | --- | --- | --- |
| Q17 | Q1 | 2.12 | 2.963 |
| Q18 | Q5 | 3.39 |
| Q19 | Q6 | 3.64 |
| Q20 | Q7 | 3.36 |
| Q21 | Q8 | 3.05 |
| Q22 | Q2 | 3.10 |
| Q23 | Q4 | 1.67 |
| Q24 | Q3 | 3.39 |
| Q25/26 | Q10 (not used in score) | |  |
| Note: one item dropped from the PAQ-C to limit respondent burden. | | | |
